# Layer-By-Layer Nanoparticle Vaccines Carrying the G Protein CX3C Motif Protect against RSV Infection and Disease

**DOI:** 10.3390/vaccines3040829

**Published:** 2015-10-12

**Authors:** Patricia A. Jorquera, Katie E. Oakley, Thomas J. Powell, Naveen Palath, James G. Boyd, Ralph A. Tripp

**Affiliations:** 1 Department of Infectious Disease, College of Veterinary Medicine, University of Georgia, 111 Carlton Street, Athens, GA 30602, USA; E-Mails: jorquera@uga.edu (P.A.J.); Katie.E.Oakley@gmail.com (K.E.O.); 2 Artificial Cell Technologies Inc., 5 Science Park, Suite 13, New Haven, CT 06511, USA; E-Mails: jpowell@artificialcelltech.com (T.J.P.); npalath@artificialcelltech.com (N.P.); jboyd@artificialcelltech.com (J.G.B.)

**Keywords:** RSV, G protein, G glycoprotein, vaccine, CX3C, immunity, nanoparticle

## Abstract

Respiratory syncytial virus (RSV) is the single most important cause of serious lower respiratory tract infections in young children; however no effective treatment or vaccine is currently available. Previous studies have shown that therapeutic treatment with a monoclonal antibody (clone 131-2G) specific to the RSV G glycoprotein CX3C motif, mediates virus clearance and decreases leukocyte trafficking to the lungs of RSV-infected mice. In this study, we show that vaccination with layer-by-layer nanoparticles (LbL-NP) carrying the G protein CX3C motif induces blocking antibodies that prevent the interaction of the RSV G protein with the fractalkine receptor (CX3CR1) and protect mice against RSV replication and disease pathogenesis. Peptides with mutations in the CX3C motif induced antibodies with diminished capacity to block G protein-CX3CR1 binding. Passive transfer of these anti-G protein antibodies to mice infected with RSV improved virus clearance and decreased immune cell trafficking to the lungs. These data suggest that vaccination with LbL-NP loaded with the CX3C motif of the RSV G protein can prevent manifestations of RSV disease by preventing the interaction between the G protein and CX3CR1 and recruitment of immune cells to the airways.

## 1. Introduction

Respiratory syncytial virus (RSV) is an important cause of severe respiratory disease in infant, young children and the elderly [1]. Severe pulmonary disease is characterized by bronchiolitis and pneumonia, which often results in abnormal lung function and is associated with asthma [2]. A characteristic feature of RSV infection is that antibody to infections does not provide lasting protection, although prior infection can modulate the severity of the disease [3,4].

RSV is a member of the *Pneumovirus* genus within the *Paramyxoviridae* family of enveloped, single-stranded, negative-sense RNA viruses. The viral genome encodes ten major mRNAs, which produce eleven viral proteins; two of these represent the major surface glycoproteins, *i.e.*, the fusion (F) and attachment (G) proteins [2]. Antibodies directed against F or G proteins neutralize virus infectivity and have a major role in protective immunity against RSV [4]. The RSV F protein induces neutralizing antibodies and a level of cross-protection against RSV strains in animal models [5,6]. Therefore, efforts to treat and prevent RSV infection have frequently focused on anti-RSV F protein antibodies, antiviral drugs that affect fusion, vector vaccines expressing F protein, or F protein-based subunit vaccines. Prophylactic treatment with palivizumab, an anti-RSV F protein neutralizing antibody has proven to be effective in preventing serious complications of RSV infection in high risk infants and young children; however, it has not been effective in treating active infection [7,8].

The RSV G protein has been implicated in pathogenesis of disease. The G protein has been associated with increased eosinophilia after RSV infection in FI-RSV vaccinated animals [1], increased pulmonary levels of Substance P after RSV challenge [1,9], Th2 bias immune response [10], and decreased respiratory rates associated with its administration to mice [11]. These responses have been attributed to the G protein CX3C chemokine motif located in the central conserved region (CCR) (amino acids 182–186) which is conserved among all RSV strains. The G protein CX3C motif binds to the CX3C chemokine receptor, CX3CR1, and mimics many activities of the only known CX3C chemokine, fractalkine [12,13]. A monoclonal antibody (mAb) that binds to the G protein CCR, mAb 131-2G, blocks G protein binding to CX3CR1, neutralizes RSV virus *in vivo* in an Fc dependent fashion, and decreases several disease manifestations in RSV challenged mice including pulmonary inflammation and mucous production [14,15,16,17,18]. A recent study compared the efficacy of mAb 131-2G with the anti-F protein mAb 143-6C, that reacts at the same antigenic site as palivizumab and has been demonstrated to reduce RSV replication and disease in animal models [19]. In this study it was demonstrated that treatment with 131-2G decreased breathing effort, mucin levels, body weight loss, and pulmonary infiltration earlier and more effectively than treatment with mAb 143-6C [19], suggesting that in mice, monoclonal antibodies directed against the G protein CX3C motif are superior for treating disease during RSV infection when compared to an anti-F protein mAb similar to Palivizumab.

Importantly, it has been shown that vaccination of mice with peptides comprising the CCR of the G protein elicit antibodies that block G protein binding to CX3CR1, and effectively cross-neutralize both A and B strains of RSV [20,21]. Vaccination with LbL nanoparticle vaccines comprising the G protein CX3C motif have also shown to induce protective neutralizing antibody response that inhibited RSV replication *in vivo*, increased T cell response to the G protein and reduced airway inflammation [22]. In the present study, we show that protection following LbL-NP vaccination requires an unmodified CX3C motif to elicit antibodies that block G protein-CX3CR1 binding. We also show that vaccination with LbL-NP encompassing the CX3C motif induces robust G protein-specific T cell responses, and antibody responses, and that both have a role in viral clearance, and protection from virus replication and airway inflammation. These findings suggest that vaccines that induce RSV G protein-CX3CR1 blocking antibodies provide an effective and safe disease intervention strategy.

## 2. Materials and Methods

### 2.1. Animals

Specific-pathogen-free, 6-to-8 weeks old female BALB/c (H-2^d^) mice (National Cancer Institute, NCI) were used in all experiments. Mice were housed in microisolator cages and were fed sterilized water and food *ad libitum*. All experiments were performed in accordance with the guidelines of the University of Georgia Institutional Animal Care and Use Committee (IACUC), with protocols approved by the University of Georgia IACUC.

### 2.2. Virus Infection

RSV A2 was propagated in Vero E6 cells (ATCC CRL-1586) as described [23]. Mice were anesthetized by intraperitoneal administration of Avertin (180–250 mg/kg; [24,25]) and intranasally challenged with 10^6^ PFU of RSV A2 in serum-free Dulbecco modified Eagle medium (DMEM; Hyclone, GE Healthcare Life Sciences, South Logan, UT, USA).

### 2.3. Peptide Synthesis

Peptides spanning the CX3C region in the CCR of the RSV G proteins from RSV strains A2, CH17 and B1 [24] were designed for the vaccine candidates (Table 1). These strains were chosen for their differences in the CX3C motif sequence. Designed peptides (DP) were obtained from Artificial Cell Technologies Inc. (New Haven, CT, USA) and they were dissolved in redox buffer (5 mM glutathione, 5 mM oxidized glutathione, phosphate pH 7) at room temperature for 6–16 h to promote folding of the CX3C motif. Folding was monitored by HPLC (>90% conversion to new peak), then DP were purified again by HPLC. Electrospray mass spectrometry showed losses of 4.0 ± 0.5 amu and the products tested sulfydryl negative in a standard DTNB assay, consistent with formation of two disulfides.

**Table 1:** RSV G_169–198_ peptides used for vaccination.

Peptide	Sequence of Designed Peptide ^a^
CX2C ^c^	NFVPCSICSNNPTCW-ICKRIPNKKPGKKT
CX3C ^b^	NFVPCSICSNNPTCWAICKRIPNKKPGKKT
CX4C ^c^	NFVPCSICSNNPTCWAAICKRIPNKKPGKKT
CX5C ^c^	NFVPCSICSNNPTCWAAAICKRIPNKKPGKKT
CH17 ^b^	NFVPCSICSNNPTCWDICKRIPNKKPGKKT
B1 ^b^	NFVPCSICGNNQLCKSICKTIPSKKPKKKP
scramble	NCTCPINKRIFACPTSIKKPNNWSGCVPKK

^a^ The location of the wild type and modified CX3C motif in the G protein is underlined. ^b^ GenBank sequences used in this study: AAC14901 (CX3C; RSV strain A2), NP_056862 (B1; RSV strain B1) and AF065255 (CH17; RSV strain NY/CH17/93). ^c^ Mutations of the wild type CX3C peptide sequence (RSV strain A2).

### 2.4. Nanoparticle Fabrication and Quality Control

Nanoparticles were constructed as previously described [26] on 50 nm diameter CaCO_3_ cores by alternately layering poly-L-glutamic acid (PGA, negative charge) and poly-L-lysine (PLL, positive charge) to build up a seven-layer film where the designed peptide (DP) containing the RSV G protein CX3C motif linked to a cationic sequence was added as the outermost layer (Table 2). The compositions of the films were determined by amino acid analysis (AAA). Endotoxin levels were measured using limulus amebocyte lysate (LAL) assay and were found to be less than 0.1 EU/µg of G peptide. The dispersity of the particle vaccines was monitored by dynamic light scattering (DLS). Stepwise LbL steadily increases the diameter of the particles several fold, from an apparent diameter of about 150 nm for uncoated particles to about 400–600 nm for fully coated particles. Some particle aggregation was detected in each batch with a second population of particles in the 1500–2000 nm range (Appendix Figure A1).

**Table 2:** Peptides used for the LbL nanoparticle vaccine fabrication.

LbL-NP	Peptide	Sequence of Designed Peptide ^a^
GA2	CX3C ^b^	NFVPCSICSNNPTCWAICKRIPNKKPGKKT(K_20_Y)
GCH17	CH17 ^b^	NFVPCSICSNNPTCWDICKRIPNKKPGKKT(K_20_Y)
GB1	B1 ^b^	NFVPCSICGNNQLCKSICKTIPSKKPKKKP(K_20_Y)

^a^ The location of the CX3C motif in the G protein is underlined. ^b^ GenBank sequences used in this study: AAC14901 (CX3C; RSV strain A2), NP_056862 (B1; RSV strain B1) and AF065255 (CH17; RSV strain NY/CH17/93).

### 2.5. Vaccination

**Figure 1:**
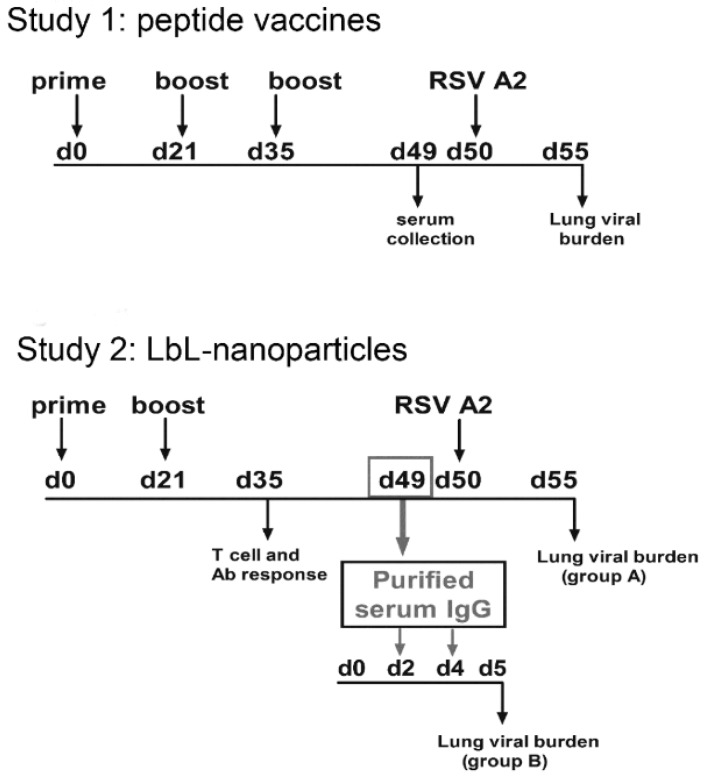
Vaccination schedule for mice studies. Experimental design for the Study 1 and Study 2.

For the peptide vaccination studies, mice were immunized by subcutaneous (s.c) administration of 20 µg of peptide diluted in 100 µL sterile phosphate buffered saline (PBS) on day 0, 21 and 35. The LbL-NP were suspended in PBS and dispersed by water bath sonication immediately prior to immunization. For the LbL NP vaccination doses were adjusted to deliver 10 µg DP/100 µL/mouse on day 0 and day 21. In both experiments (Figure 1, Studies 1 and 2) mice were s.c. immunized without adjuvant between the shoulder blades. For Study 2, the control groups received 100 µL of PBS per injection (negative control).

A sample size of *n* = 5 mice per group was used in Study 1 and *n* = 4 in Study 2. Both studies were repeated three times to ensure repeatability.

In Study 1 (*n* = 5) mice were immunized by subcutaneous (s.c) administration of 20 µg of peptide diluted in 100 µL PBS on day 0, 21 and 35. In Study 2 (*n* = 4) the LbL-NP were suspended in PBS to deliver 10 µg DP/100 µL/mouse on day 0 and day 21 and the control groups received 100 µL of PBS per injection (negative control). Mice sera from Study 2 (group A) was collected on day 49 post vaccination and passively transfer to RSV-infected mice on day 2 and 4 post infection (group B). Both studies were repeated three times to ensure reproducibility.

### 2.6. Purification of Total IgG and Passive Transfer of Antibodies

Total IgG was purified from mouse immune sera collected at day 49 post vaccination (Figure 1, Study 2) or from naïve mice with the NAbTM Protein G Spin kit (Thermo Fisher Scientific, Grand Island, NY, USA) following the procedure recommended by the manufacturer. Purified IgG was dialyzed against PBS (pH 7.2), concentrated using a Centricon spin column (Millipore, Billerica, MA, USA) with a 30-kDa cut-off and filter sterilized (0.45 micron, Millipore) for injection. After purification, IgG was tested by indirect ELISA to confirm binding to RSV A2 as describe below. The protein concentrations were determined by BCA protein assay (Pierce Protein Research Products) [27].

Mice were challenged by intranasal (i.n.) administration of 10^6^ PFU RSV A2 on day 0 and intraperitoneally (i.p.) treated with 250 μg mAb 131-2G, purified mouse IgG from mice vaccinated with LbL-NP (Study 2, group B) or normal mouse serum on day 2 and 4 post-infection (p.i.). On day 5 p.i. mice were euthanized and lungs and bronchioalveolar lavages (BAL) were collected for further analysis.

### 2.7. Indirect ELISA

Serum IgG antibodies to G peptide 169–198 (G_169–198_) and RSV A2 were detected by ELISA using 96-well high binding plates (Corning, New York, NY, USA) coated with 20 µg/mL of G_169–198_ or 10^6^ PFU/mL RSV A2 in 0.05 M carbonate-bicarbonate buffer, pH 9.6. Sera were added to plates in serial dilutions and naïve mouse serum was used as negative control. G peptide- and RSV-specific antibodies were detected with horseradish peroxidase (HRP) conjugated antibodies specific for mouse IgG, IgG1 or IgG2a (Southern Biotech, Birmingham, AL, USA) followed by addition of SureBlue TMB 1-Component Microwell Peroxidase Substrate (KPL, Inc., Gaithersburg, MD, USA) and TMB stop solution (KPL, Inc.). Antibody titers were determined as the last sample dilution that generated an OD_450_ reading of greater than 0.2. This cut-off value was determine based on the mean OD value of naïve mouse serum + 3 × standard deviation of the mean.

### 2.8. G Protein-CX3CR1 Binding Inhibition Assay

Total IgG was purified from sera of vaccinated mice using immobilized protein G (Thermo Scientific) according to the manufacturer’s protocol. To evaluate the ability of RSV G protein peptide-specific antibodies to prevent RSV G protein binding to CX3CR1, 1 µg of purified serum IgG antibody was incubated with 1 µM of native RSV A2 G protein for 1 h at 4 °C. IgG purified from naive mouse serum was used as negative antibody control, and the monoclonal antibody (mAb) 131-2G was used as positive antibody control in all the assays. 293-CX3CR1^+^ cells and untransfected 293 cells were plated in a round-bottom 96-well plate at 2 × 10^5^ cells per well, washed with PBS, and incubated with PBS containing anti-human CD32 (Fc block; Millipore) at 1 µg/mL and 4 °C for 15 min. After incubation, the cells were resuspended in a pre-incubated mixture of purified IgG and native RSV G protein, and 5 µg/mL of heparin (Sigma-Aldrich, St. Louis, MO, USA) was added to prevent any nonspecific binding, and incubated for 1 h at 4 °C. After the incubation, the cells were washed in PBS containing 1% bovine serum albumin (BSA) and incubated with mAb 130-2G conjugated to Alexa-Fluor 488 (AF488; Molecular Probes, Eugene, OR, USA) for 30 min at 4 °C. The percentage (%) of G-protein binding to 293-CX3CR1^+^ or 293 cells only was determined by flow cytometry using a BD LSRII and FlowJo analysis software (Tree star Inc., Ashland, OR, USA, 1996). The percent inhibition was calculated using the formula: 1-[(% AF488^+^ 293-CX3CR1^+^ cells treated with G protein + antibody mixture) / (% AF488^+^ 293-CX3CR1^+^ cells treated with G protein)].

### 2.9. Lung Virus Titers

RSV lung virus titers in vaccinated and control mice were determined as previously described [23]. Briefly, lungs were aseptically removed from mice at day 5 post-RSV challenge (10^6^ PFU/mouse), and individual lung specimens were homogenized at 4 °C in 1 mL of serum-free DMEM (Hyclone) by use of gentleMACS™ Dissociator (Miltenyi Biotec Inc., San Diego, CA, USA). Samples were centrifuged for 10 min at 200× *g* the supernatants were transferred to a new tube and used immediately or stored at −80 °C until they were assayed. For the plaque assay, 10-fold serial dilutions of the lung homogenates were added to 90% confluent Vero E6 cell monolayers. Following adsorption for 2 h at 37 °C, cell monolayers were overlaid with 2% methylcellulose media and incubated at 37 °C for 6 days. The plaques were enumerated by immunostaining with monoclonal antibody 131-2A against RSV F protein followed by a secondary goat anti-mouse IgG antibody conjugated to alkaline phosphatase (AP) (Thermo Fisher Scientific, Grand Island, NY, USA). Plaques were developed using 200 µL/well of 1-Step^TM^*NBT*/*BCIP* (Thermo Scientific) at room temperature for 15–30 min. Plaques were counted using a dissecting microscope.

### 2.10. ELISPOT Analysis

96-well Multiscreen plates (Millipore) were coated with either 1 µg/mL anti-mouse IL-4 or anti-mouse IFN-γ capture antibody (R&D Systems, Minneapolis, MN, USA) and incubated overnight at 4 °C. The plates were then blocked by the addition of 200 µL of RPMI-10 medium (RPMI 1640 supplemented with 10% FBS, 100 U/mL penicillin, 100 µg/mL streptomycin, 50 µM 2-mercaptoethanol and 2 mM L-glutamine) and incubated for 2 h at 37 °C. In parallel, spleens were harvested from vaccinated and naïve mice at day 35 post vaccination and prepared to a single cell suspension using a syringe plunger and a 70 µm mesh nylon strainer. The cell suspensions were collected by centrifugation for 10 min at 200× *g* and suspended in RPMI-10 at a concentration of 107 cells/mL. A total of 1 × 105, 5 × 105 and 1 × 106 spleen cells were added to each well, and cells were stimulated with either 5 µg/mL G183–197 (WAICKRIPNKKPGKK) or 5 µg/mL eGFP200-208 (HYSTQSAL) (irrelevant peptide control) for 24 h at 37 °C and 5% CO_2_. Plates were washed 4 times with wash buffer (0.05% Tween-20 in PBS), anti-mouse IL-4 or anti-mouse IFN-γ detection antibody (R&D Systems) was added and plates were incubated overnight at 4 °C. Detection antibody was removed, plates were washed and cytokine spots were developed using ELISpot blue color module (R&D Systems). Spots were counted using an ELISPOT reader (AID EliSpot Reader System, San Diego, CA, USA). RSV-specific ELISPOT numbers were determined from triplicate wells/cell population by subtracting the mean number of ELISPOTs in the eGFP200-208 stimulated wells and the data is presented as IL-4 or IFN-γ spots per every 1 × 10^6^ spleen cells.

### 2.11. BAL Cell Collection

Five days post-infection, a subset of mice from each group was sacrificed and a tracheotomy was performed to access bronchoalveolar lavage (BAL). The mouse lungs were washed three times with 1 mL of cold PBS and the retained BAL was centrifuged at 400× *g* for 5 min at 4 °C. Cell pellets were resuspended in 200 µL of FACS staining buffer (PBS containing 1% BSA), BAL cell were counted using a hemocytometer (Thermo Fisher Scientific, Grand Island, NY, USA) and cells were stained and analyzed by flow cytometry as described below.

### 2.12. Flow Cytometry

Cell suspensions were blocked with FcγIII/II receptor antibody (BD), and subsequently stained with antibodies from BD bioscience (San Jose, CA, USA), *i.e.*, PerCP-Cy5.5-conjugated anti-CD45 (30-F11), FITC-conjugated anti-CD11c (HL3), PE-conjugated anti-SiglecF (E50-2440), PE-Cy7 conjugated anti-Gr-1 (cloneRB6-8C5), APC conjugated anti-CD3e (145-2C11). Cells were acquired on a LSRII flow cytometer (BD bioscience) with data analyzed using FlowJo software (v7.6.5, Tree Star Inc., Ashland, OR, USA). Cell surface marker expression patterns were used to identify the following cell types: T cells (CD45^+^ CD3^+^), neutrophils (CD45^+^ Gr-1^high^), eosinophils (CD45^+^ SiglecF^+^ CD11c^low^), and alveolar macrophages (CD45^+^SiglecF^+^CD11c^high^) [28,29,30].

### 2.13. Statistics

All statistical analyses were performed using GraphPad software (San Diego, CA, USA). Statistical significance was determined using One-way ANOVA followed by Bonferroni’s *post-hoc* comparisons tests, a *p* value < 0.05 was considered significant.

## 3. Results

### 3.1. Vaccination with Candidates Having Modified G Protein CX3C Motifs Induce Antibodies that Bind Poorly to the RSV G Protein

It has been previously shown that mice immunized with the G protein peptides consisting of the central conserved region (CCR) of the RSV G protein generate antibodies that block binding of the G protein to CX3CR1 and neutralize both A and B strains of RSV [20,21,31]. Vaccination with LbL-NP consisting of the G protein 169–198 amino acid region induced protection in mice challenged with RSV [22]. In these studies it was shown that vaccination with the G protein CX3C motif protected against RSV and disease pathogenesis by inducing antibodies that block the interaction between the G protein and CX3CR1.

**Figure 2:**
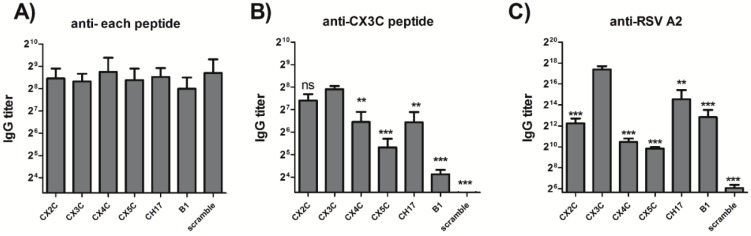
Antibody response to peptide vaccination. Serum IgG was measured in the serum of vaccinated animals at day 49 post vaccination (Study 1, *n* = 5). IgG against each respective G peptide (**A**), CX3C peptide (**B**) or RSV A2 (**C**) were detected by indirect ELISA assay. Experiments were independently repeated at least 3 times and analyzed by one-way ANOVA test. Ns, no significant; ** *p* <0.005; *** *p* <0.0005 *vs*. CX3C peptide.

To better understand the importance of the CX3C motif in mediating antibody response against the RSV G protein, mice were immunized with peptides carrying CX2C, CX3C, CX4C or CX5C motifs (Table 1). Mice were vaccinated on days on day 0, 21 and 35 with 20 µg of peptides, and antibody responses were measured on day 49 (Figure 1, Study 1). All peptide vaccines elicited similar IgG response against the specific peptide used for vaccination (Figure 2A), indicating the peptides were similar immunogens. Mice vaccinated with the G_169–189_ peptides, but not the scramble peptide developed serum IgG that reacted to the wild type CX3C peptide (Figure 2B), as well as the G protein expressed on RSV particles (Figure 2C). However, mice vaccinated with the wild type CX3C peptide showed a stronger IgG response against the CX3C peptide and RSV (Figure 2B,C). Removing (CX2C) or adding amino acid residues (CX4C and CX5C) between the cysteines 182 and 186 had a detrimental effect on the G protein-specific antibody response (Figure 2B,C). Similarly, changes within the CX3C motif (CH17 and B1 peptides) and outside the CX3C motif (B1 peptide) also affected the specificity of the antibodies for G protein CCR, since antibodies from mice vaccinated with the CH17 and B1 peptides showed lower anti- CX3C peptide specificity compared to the group vaccinated with the wild type CX3C peptide (Figure 2B). Interestingly, vaccination with the CX3C, CH17 and B1 protein peptides induced higher IgG titers against RSV A2 than vaccination with CX2C, CX4C and CX5C, suggesting that mutations within the CX3C motif that modify the distance between Cys182 and Cys186 may affect the folding of these peptides inducing the formation of a different epitope, and thus antibodies directed against these new regions are no longer able to bind to the wild type CX3C motif and likely unable to inhibit the G protein-CX3CR1 interaction.

### 3.2. Vaccinations with Candidates Having Modified CX3C Motifs Induces Antibodies That Do Not Block G Protein-CX3CR1 Binding

**Figure 3:**
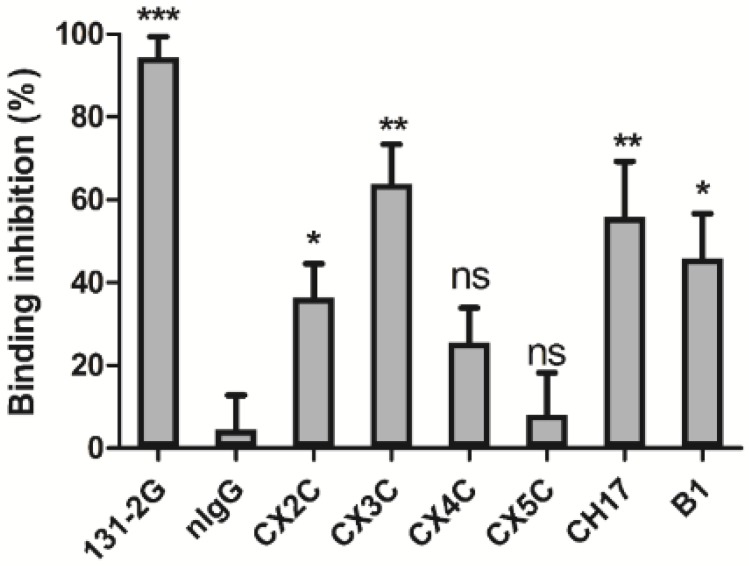
RSV G protein CX3C-CX3CR1 blocking antibody studies. Total IgG was purified from serum derived from mice vaccinated with CX3C peptides and evaluated for the ability to prevent native RSV G protein binding to CX3CR1 expressed on HEK293-CX3CR1 cells. Data are presented as the percent inhibition of RSV G protein binding to HEK293-CX3CR1 cells. Ns, non-significant; * *p* < 0.05; ** *p* <0.005; *** *p* < 0.0005 compared to nIgG (one-way ANOVA, *n* = 5).

**Figure 4:**
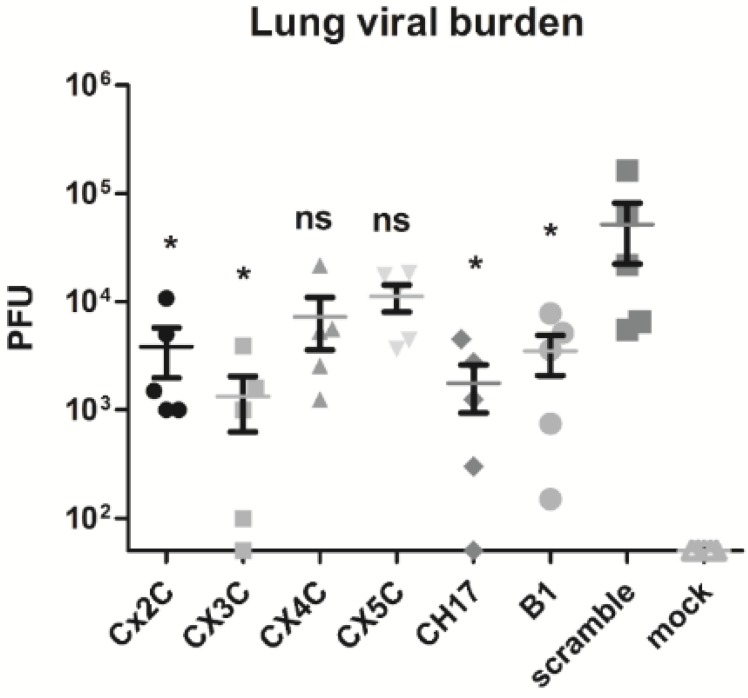
Lung virus titers following RSV challenge of mice vaccinated with peptides. Mice were vaccinated subcutaneously (s.c) with CXC3 peptides or scramble control peptide, challenged i.n. with 10^6^ PFU RSV A2, and lung virus titers were determined at day 5 post-challenge. Lung virus titers were determined by immunostaining plaque assay. Data are presented as total PFU/whole lung tissue. Experiments were independently repeated at least 3 times and analyzed by one-way ANOVA test. Values shown as the mean ± SEM; * *p* < 0.05 *vs*. scramble peptide.

To test the functionality of the antibodies that were induced by the vaccine candidates and their ability to prevent G protein binding to CX3CR1, purified serum IgG from mice vaccinated with G peptide candidates was examined in a G protein-CX3CR1 binding inhibition assay. The positive control, mAb 131-2G directed against the G protein CX3C motif [14,16,17,18], almost totally inhibited the G protein binding to the fractalkine receptor; CX3CR1 (Figure 3). Similarly, strong inhibition of binding was observed for total serum IgG from mice vaccinated with the CX3C (63.9%) and CH17 (55.9%) peptides, and somewhat less so for IgG from B1 (45.8%) vaccinated mice (Figure 3). Serum IgG from mice vaccinated with the mutant CX3C peptides showed reduced inhibition [CX2C (36.4%) > CX4C (25.6%) > CX5C (8.1%)] compared to the wild type CX3C peptide (63.9%) (Figure 3), suggesting that these antibodies are less efficient blocking the G protein CX3C motif and preventing RSV infection. To determine whether these results impacted efficacy of the vaccine peptides, mice were challenged at day 50 post-vaccination with 10^6^ PFU of RSV and the lung viral burden was determined at day 5 post-infection (pi) (Figure 1, Study 1). Consistent with the previous data, mice vaccinated with CX3C and CH17 peptides, followed by B1, showed the lowest lung viral burden among the vaccinated groups, and mice vaccinated with the mutants CX3C motifs showed limited (CX2C peptide) or no significant (*p* < 0.05) reduction (CX4C and CX5C peptides) in viral replication (Figure 4). Taken together, these results indicate that during vaccination the G protein CX3C motif is required to induce antibodies that protect against the G protein-CX3CR1 binding and RSV replication.

### 3.3. LbL-NP Carrying the G Protein CX3C Motif Induce Strong Humoral and T Cell Response That Protect against RSV Infection

**Figure 5:**
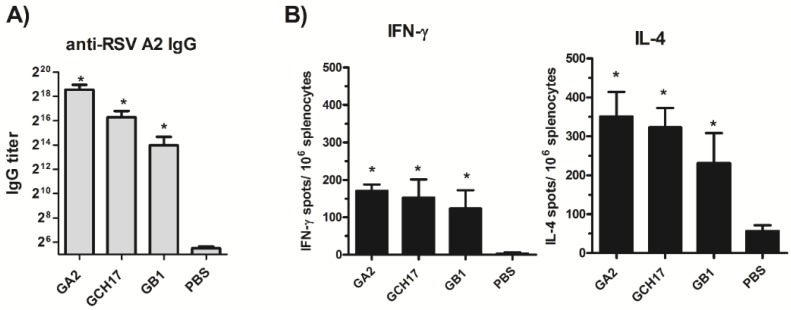
Humoral and cellular responses induced by LbL nanoparticle vaccination. Mice were vaccinated with LbL nanoparticles on day 0 and 21. At day 35 post vaccination serum was collected and RSV-specific IgG response was measured via indirect ELISA assay (**A**). At the same time point spleens were harvested, splenocytes were stimulated with G peptide 183–197 for 24 h and IFN-γ and IL-4 secretion detected by ELISPOT assay (**B**). Experiments were independently repeated at least 3 times and analyzed by one-way ANOVA test. * *p* <0.05 compared to PBS, values are shown as the mean ± SEM.

**Figure 6:**
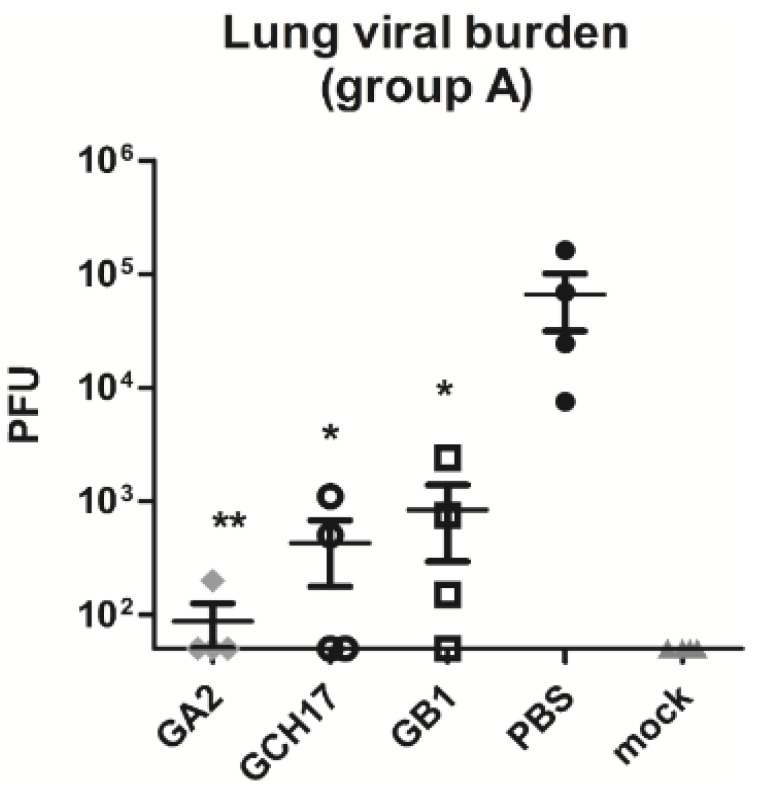
Lung virus titers following RSV challenge of mice vaccinated with LbL nanoparticles. Mice were vaccinated s.c. with LbL nanoparticles carrying the G protein Cx3C motif, challenged i.n. with 10^6^ PFU RSV A2, and lung virus titers were determined at day 5 post-challenge. Lung virus titers were determined by immunostaining plaque assay. Data are presented as total PFU/whole lung tissue. Values shown as the mean ± SEM (one-way ANOVA); * *p* <0.05; ** *p* < 0.005 *vs*. PBS vaccinated group.

**Figure A2:**
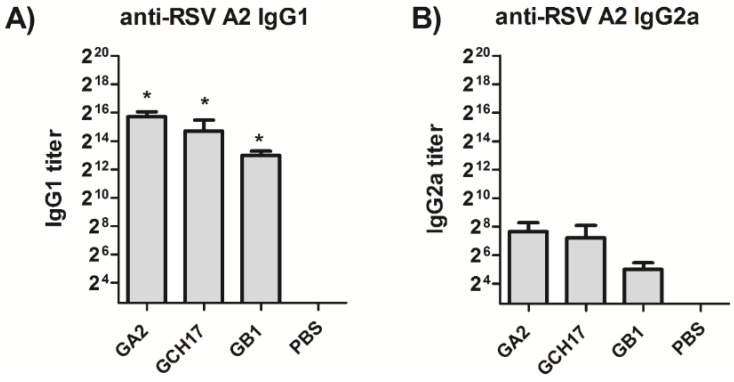
Humoral response induced by LbL nanoparticle vaccination. Mice were vaccinated with LbL nanoparticles on day 0 and 21. At day 35 post vaccination serum was collected and RSV-specific antibody isotypes IgG1 (**A**) and IgG2a (**B**) levels were measured via indirect ELISA assay. Experiments were independently repeated at least 3 times and analyzed by one-way ANOVA test. * *p* < 0.05 compared to PBS, values are shown as the mean ± SEM.

The Layer-by-Layer (LbL) nanoparticle vaccine platform was utilized to develop nanoparticle vaccines carrying CX3C peptides. The peptides chosen for vaccine production were CX3C, CH17 and B1 because these three peptides showed similar measures of protection against RSV A2 (Figure 3 and Figure 4). Mice were vaccinated with 10 µg of LbL-NP at days 0 and 21, and the immune response to vaccination was determined on day 35 (Figure 1, Study 2). Similar to the peptide vaccination, mice vaccinated with the GA2 (CX3C peptide), GCH17 (CH17 peptide) and GB1 (B1 peptide) LbL-NP developed strong serum IgG response against the RSV G protein (Figure 5A). Vaccination with LbL-NP induced both IgG1 and IgG2a isotypes, with IgG1 being more predominant then IgG2a (Appendix Figure A2). Immunization with nanoparticles also induced a RSV G protein-specific T cell response characterized by the expression of IFN-γ and IL-4 upon G_183–197_ peptide stimulation, indicating that nanoparticle vaccination induces Th1 and Th2 response (Figure 5B). In general, the strength of the humoral and T cell response against the RSV A2 G protein induced by nanoparticle vaccination followed the same trend and hierarchy where GA2 > GCH17 > GB1. To determine if the strength of the immune response correlated with protection against RSV replication, mice were i.n. challenged with 10^6^ PFU of RSV and the lung viral burden was determined at day 5 p.i. As shown in Figure 6, all LbL nanoparticle vaccinated groups showed reduced lung viral burden compared to the saline control group. The GA2 nanoparticle group had the lowest lung viral titer, followed by GCH17 and GB1, demonstrating that GCH17 and GB1 induce cross-protection against RSV A2. Taken together, these results demonstrate that vaccination with LbL nanoparticles having the amino acid region 169–189 of the RSV G protein induce humoral and T cell response that protects against RSV replication.

### 3.4. Antibodies Induced by G Protein CX3C Nanoparticles Vaccination Protect against RSV Replication and Disease Pathogenesis

**Figure 7:**
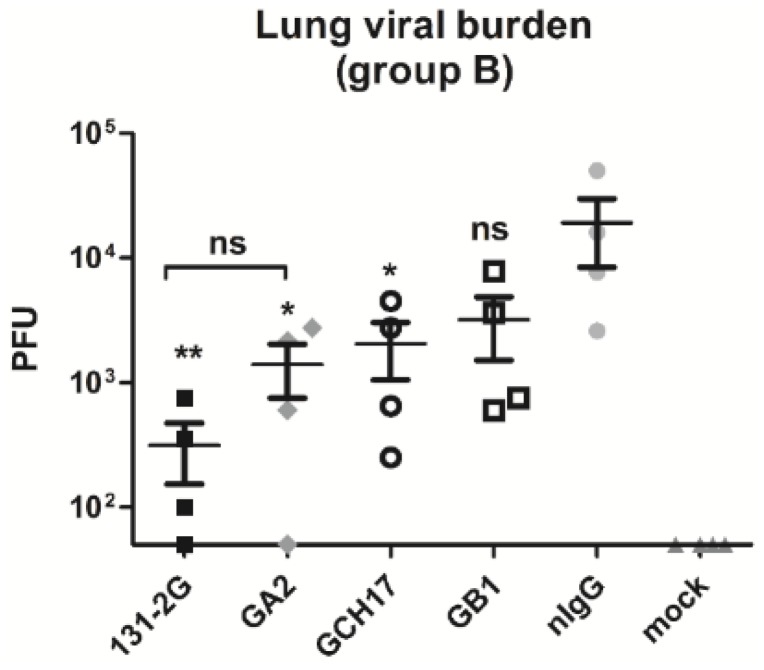
Lung virus titers following RSV challenge of mice passively transferred with purified IgG from mice vaccinated with LbL nanoparticles. Mice were challenged i.n. with 10^6^ PFU RSV A2, and passively transferred with purified IgG from group A (Study 2) on day 2 and 4 post challenge. At day 5 post challenge lung virus titers were determined by immunostaining plaque assay. Data are presented as total PFU/whole lung tissue. Values shown as the mean ± SEM (one-way ANOVA); ns, non-significant; * *p* < 0.05; ** *p* < 0.005 *vs*. nIgG group.

Antibodies induced by vaccination with CX3C, CH17 and B1 peptides inhibited the G protein binding to the fractalkine receptor (Figure 3), and it was previously shown that anti-G protein CX3C antibodies protect against RSV replication and pathogenesis [14,20]. Thus, the LbL nanoparticle vaccine candidates were tested for their ability to induce antibodies that provided protection against RSV infection and disease. Total serum IgG from mice vaccinated with GA2, GCH17 and GB1 nanoparticles was purified at day 49 post immunization, and passively transferred to BALB/c mice infected with RSV A2 at day 2 and 4 post challenge (Figure 1, Study 2: group B). At day 5 pi lung tissues were harvested and viral titers were measured by plaque assay. As seen in Figure 7, passive transfer of the positive control mAb 131-2G significantly (*p* < 0.05) reduced RSV replication as one might predict [14,18,19,32]. Therapeutic i.p. administration of purified IgG from GA2-serum and GCH17-serum significantly (*p* < 0.05) reduced RSV lung replication compared to normal IgG control (nIgG). Purified IgG from GB1-serum moderately reduced viral replication; however, the reduction was not statistically significant (Figure 7). These results indicate that antibodies directed against the G protein CX3C motif can efficiently neutralize RSV *in vivo* and increase viral clearance.

**Figure 8:**
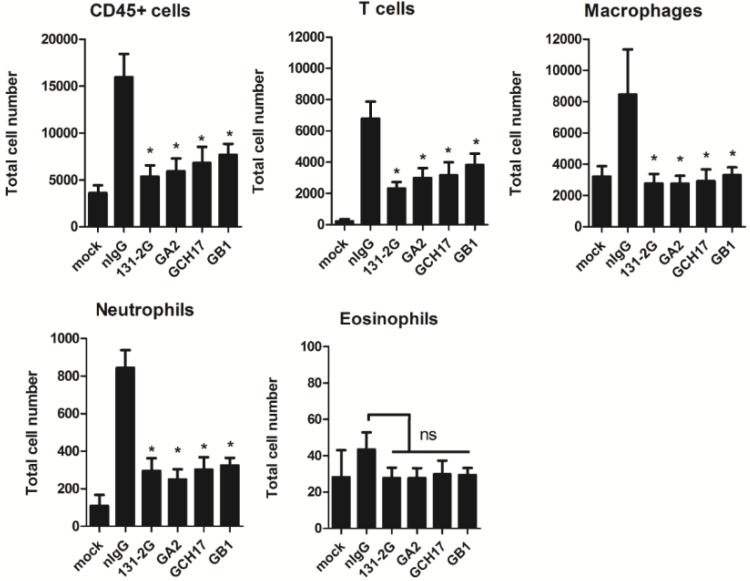
Pulmonary infiltration following therapeutic antibody treatment. BAL leukocytes (CD45^+^ cells), T cells (CD45^+^ CD3^+^), Macrophages (CD45^+^ SiglecF^+^ CD11c^+^), Neutrophils (CD45^+^ Gr1^+^) and Eosinophils (CD45^+^ SiglecF^+^ CD11c-) numbers in the lungs of mice treated at day 2 and 4 p.i. with nIg, anti-RSV G mAb 131-2G or purified IgG from LbL nanoparticle were determined at day 5 p.i. Data shown are the means ± SEM. Asterisks indicate a significant difference (*p* < 0.05) between nIgG-treated and antibody treated mice.

It has been previously shown that treatment of RSV infected mice with the mAb 131-2G can reduce pulmonary inflammation [14,19]. Thus it was important to determine if antibodies elicited by vaccination with LbL nanoparticles vaccine candidates could also reduce leukocyte trafficking in the lungs. As shown in Figure 8, mice treated with purified IgG from LbL nanoparticle vaccinated mice have reduced numbers of pulmonary leukocytes, specifically T cells, macrophages and neutrophils. No differences in the number of eosinophils were detected in mice following therapeutic treatment.

Taken together, these results demonstrate that vaccination with LbL-NP carrying the G protein Cx3C motif elicits humoral and cellular responses that protect animals from RSV infection. Moreover, the antibodies induced by these nanoparticles vaccine candidates have properties similar to te mAb 131-2G, *i.e.*, they facilitate RSV virus lung clearance, and they reduce pulmonary infiltration, an effect that protects mice from RSV infection and disease. The results demonstrate that a RSV G protein vaccine candidate consisting of G protein peptide derived from CCR region 169–198 of the G protein CX3C motif will induce antibodies that block G protein CX3C-CX3CR1 interaction, inhibit RSV replication and disease, and provide a new, safe, and efficacious RSV vaccine strategy.

## 4. Discussion

The RSV G protein central conserved region (CCR) contains a CX3C chemokine motif that is highly conserved among RSV species [12,20]. The CX3C motif binds to the CX3C chemokine receptor (CX3CR1) and mimics activities of the only known CX3C chemokine, fractalkine [11,12,33].This motif is located between aa 182–186 of the G protein and includes two of the four evolutionarily conserved cysteines at aa 173, 176, 182, and 186 which form a cysteine noose structure.

In the current study, the importance and role of the RSV G protein CX3C motif in the induction of protective antibodies was evaluated in mice vaccinated with G protein peptides having mutant versions of the CX3C motif. Antibodies directed against the modified CX3C peptides (CX2C, CX4C and CX5C) were able to bind to the CX3C peptide and to RSV virions (Figure 2), but they minimally blocked the G protein CX3C binding to CX3CR1 (Figure 3) indicating that vaccination with the modified peptides elicited antibodies that bound to the CCR but outside the CX3C motif. Though the antibodies bound G protein, they failed to effectively prevent G protein-CX3CR1 interaction and were therefore less efficient at neutralizing RSV and promoting viral clearance from the lungs (Figure 4). Vaccination with the G169–198 CX3C peptides from CH17 and B1 induced higher levels of CX3C-blocking antibodies (Figure 3) than vaccination with CX2C, CX4C and CX5C. This finding suggests that the CX3C motif is critical in inducing blocking antibodies, and that modifying the distance between Cys182 and Cys186 may affect the structure or antigenicity of the CX3C motif region, thus antibodies directed against the modified epitope cannot effectively bind to the wild type CX3C motif and are unable to inhibit the binding of the G protein to CX3CR1. A recent study demonstrated that infection of human epithelial cells with a mutant RSV CX4C virus induced higher levels of interferon type I/III and TNF-α than a wild type CX3C virus, and the level of these cytokines was similar to the level induced by treatment of RSV-infected cells with mAb 131-2G, suggesting that the CX4C mutation reduces binding to CX3CR1 [34]. Similarly, it was shown that treatment of BALB/c mice with purified G protein reduced respiratory rates, while mutated G glycoprotein lacking the CX3C motif showed no reduction in respiratory rates, and a G protein with a the CX4C mutation slightly decreased the respiratory rates, where it was found that the addition of an Ala residue to the CX3C motif reduced binding to CX3CR1 [11]. These data combined with the current results support the notion that intact CX3C motif is required for the G protein binding to CX3CR1 and for the induction of CX3C-specific antibodies during vaccination, thus only peptides containing the intact CX3C motif were chosen for vaccine development.

Layer-by-Layer (LbL) vaccine candidate technology has applications in a number of medical fields, depending on the characteristics of the core substrate used in this technology that can produce cell- or virus-like particles suitable for the delivery of biomolecules. By incorporating defined peptides having linear epitopes in LbL nano- and microparticle vaccines, investigators have demonstrated improved immunogenicity of B and T cell epitopes in diverse model systems [26,35]. Recently, our group showed that vaccination of BALB/c mice with nanoparticles [22] and microparticles comprising the G protein CX3C chemokine motif elicited strong G protein-specific T cell responses, and a neutralizing antibody response that inhibited RSV replication in the lungs of mice. Consistent with these and other related findings, the results showed that vaccination with GA2, GCH17 and GB1 LbL-NP induced strong antibody and T cell responses against the RSV G protein (Figure 5) that protected (and cross-protected) mice from RSV replication in the lung (Figure 6). It is important to note that delivery of the CX3C peptide in the LbL-NP vaccine format increased the peptide immunogenicity and reduced the dose of vaccine require to achieve protection, without the need for exogenous adjuvants or complex formulations. Specifically, mice vaccinated with LbL-NP received only two injections of 10 µg peptide and had much lower lung viral titers post-challenge (Figure 6) than mice vaccinated with three injections of 20 µg soluble peptide (Figure 4). It is known that peptides can be poor immunogens due in part to limitations that include a lack of appropriate T cell help, poor induction of CD8 T cell response and the lack of surface Ig receptor clustering required for activation of resting B cells [36]. In contrast, the increased potency of nanoparticle vaccine constructs has been attributed to mechanisms including efficient phagocytosis of the particles, antigen cross-presentation, and activation of dendritic cells and macrophages by increased cytokine production and co-stimulatory marker expression [37,38,39,40,41,42]. It is likely that upon vaccination the LbL-NP are recognized by antigen-presenting cells such as dendritic cells or macrophages and then transported to the draining lymph node, where appropriate antigen presentation occurs [43].

It has been previously shown that monoclonal antibodies that target the G protein CX3C motif have both anti-viral and anti-inflammatory effects that effectively treat RSV disease [18,19,32]. Here it was demonstrated that by vaccinating mice with LbL-NP comprising the G protein CX3C motif it is possible to induce antibodies that protect against RSV replication and lung inflammation. This is emphasized here by the protection provided to naïve mice following passive administration of antibody harvested from mice immunized with LbL-NP (Figure 7). Also, the reduced pulmonary cell infiltration in the recipient mice was similar to that seen in mice treated with mAb 131-2G (Figure 8). MAb 131-2G blocks G CX3C protein binding to CX3CR1, neutralizes RSV *in vivo* in an Fc dependent fashion [14,15,16] and decreases several disease manifestations in RSV challenged mice including pulmonary inflammation and mucous production, increased airway resistance after primary infection in mice, and enhanced inflammation in RSV-challenged FI-RSV vaccinated mice [14,15,18]. Treatment with mAb 131-2G decreases pulmonary inflammation more effectively than an anti-F mAb, 143-6C, that reacts at the same antigenic site as palivizumab, and like palivizumab both neutralizes RSV and inhibits RSV fusion [19]. These data shows the LbL-NP loaded with G CX3C peptide are good RSV vaccine candidates that elicit antibodies with similar properties to the mAb 131-2G, and are safe and effective RSV candidate vaccines.

Taken together, our data suggest that the G protein CX3C motif should be included in a G protein-based vaccine to elicit CX3C-blocking antibodies, and this can be achieved using the LbL assembly technology. Nanoparticles or microparticles carrying the G protein CX3C 169–198 region induce protection from RSV infection and disease, and elicit antibodies that lead to reduction in viral load and diminished pulmonary infiltration, protecting mice from RSV infection and disease.

## 5. Conclusions

The results from this study demonstrate that the G protein 169–198 region should be included in a G protein-based RSV vaccine to elicit CX3C-blocking antibodies and prevent RSV disease. Using the LbL vaccine assembly technology it is possible to generate nanoparticles carrying the G protein CX3C motif which induce protection from RSV infection and disease in mice, and elicit antibodies that reduce the viral load and diminish pulmonary leukocyte infiltration. The results show that LbL nanoparticles vaccines comprising region 169–198 of the RSV G protein may offer a new, safe, and efficacious RSV vaccine strategy.
